# Three annotated chromosome-level de novo genome assemblies of *Lomentospora prolificans* provide evidence for a chromosomal translocation event

**DOI:** 10.1093/g3journal/jkaf091

**Published:** 2025-04-24

**Authors:** Nina T Grossman, Yunfan Fan, Aleksey V Zimin, Maggie P Wear, Anne Jedlicka, Amanda Dziedzic, Livia C Liporagi-Lopes, Winston Timp, Arturo Casadevall

**Affiliations:** Department of Molecular Microbiology and Immunology, Johns Hopkins Bloomberg School of Public Health, Johns Hopkins University, 615 N. Wolfe St., Baltimore, MD 21205, United States; Department of Biomedical Engineering, Johns Hopkins University, 3400 N. Charles Street, Baltimore, MD 21218, United States; Department of Biomedical Engineering, Johns Hopkins University, 3400 N. Charles Street, Baltimore, MD 21218, United States; Department of Molecular Microbiology and Immunology, Johns Hopkins Bloomberg School of Public Health, Johns Hopkins University, 615 N. Wolfe St., Baltimore, MD 21205, United States; Department of Molecular Microbiology and Immunology, Johns Hopkins Bloomberg School of Public Health, Johns Hopkins University, 615 N. Wolfe St., Baltimore, MD 21205, United States; Department of Molecular Microbiology and Immunology, Johns Hopkins Bloomberg School of Public Health, Johns Hopkins University, 615 N. Wolfe St., Baltimore, MD 21205, United States; Department of Molecular Microbiology and Immunology, Johns Hopkins Bloomberg School of Public Health, Johns Hopkins University, 615 N. Wolfe St., Baltimore, MD 21205, United States; Department of Biomedical Engineering, Johns Hopkins University, 3400 N. Charles Street, Baltimore, MD 21218, United States; Department of Molecular Biology and Genetics, Johns Hopkins University, 725 N. Wolfe Street, Baltimore, MD 21205, United States; Department of Molecular Microbiology and Immunology, Johns Hopkins Bloomberg School of Public Health, Johns Hopkins University, 615 N. Wolfe St., Baltimore, MD 21205, United States

**Keywords:** *Lomentospora prolificans*, genome assembly, chromosomal translocation, subtelomere

## Abstract

*Lomentospora prolificans* is a fungal pathogen responsible for serious, often fatal, illness in patients with compromised immune systems. Treatment is rarely successful because *L. prolificans* is inherently resistant to all major classes of antifungal drugs. In this study, we publish 3 chromosome-level de novo genome assemblies, including the first complete-level assembly of *L. prolificans*, along with genome annotations. The *L. prolificans* genome is packaged in 11 nuclear chromosomes and 1 mitochondrial chromosome, has 36.7–37.1 Mb, and encodes for a putative 7,357–7,640 genes. The length and composition of contigs in 1 strain varied from those of the other 2 strains, supporting the hypothesis that a chromosomal translocation took place. These assemblies were confirmed with pulsed-field gel electrophoresis. The availability of more complete genomes will hopefully help the search for new antifungal drugs and provides insights into the evolutionary history of this pathogenic fungus.

## Teaser

*Lomentospora prolificans* is a fungal pathogen responsible for serious, often fatal, illness in patients with weakened immune systems. In this study Grossman *et al*. publish three genome assemblies of *L. prolificans*, along with the proteins believed to be transcribed from these genomes. The genome consists of 11 nuclear chromosomes and one mitochondrial chromosome, and encodes for more than 7300 genes. The contents of the genome of one of the three sequenced strains were organized differently from the other two, suggesting that at some point portions of this strain's chromosomes underwent a chromosomal translocation.

## Introduction

*Lomentospora prolificans* is a filamentous fungal pathogen that causes disease primarily in severely immunocompromised patients, most often those with hematological malignancy and neutropenia (Jacobs *et al*. 2020). Though rare relative to other fungal diseases, these infections are very difficult to treat with current antifungal therapy, and, accordingly, disseminated infections have a mortality rate of 80% (Jenks *et al*. 2020). The reason for this high frequency of antifungal treatment failure is that *L. prolificans* is intrinsically resistant to all 3 major families of systemic antifungal agents: azoles, echinocandins, and polyenes.

Thus far, relatively little research has been conducted on the causes for this remarkable drug resistance, and of the 3 drug families, a mechanism of resistance has been proposed for only echinocandins. One major reason for this dearth of information is that, until very recently, no whole genome assembly was available for this organism. This changed in 2017 when Luo *et al*. published the first whole genome assembly of clinical *L. prolificans* isolate JHH-5317 (Luo *et al*. 2017). Sequenced with Illumina and Oxford Nanopore systems, this assembly consisted of 1,625 contigs, of which 26 contained 98% of the total length of 37.63 Mb.

This whole genome sequence represents a massive resource for researchers wishing to study *L. prolificans* and has already informed work on its mechanisms of resistance (Wu *et al*. 2020). However, in its current fragmented state and deriving from a single strain, the representativeness of the existing genomic data is unknown and this single genome sequence is an imperfect foundation upon which to build the entirety of *L. prolificans* molecular biology.

To generate a more complete, chromosome-level assembly of the *L. prolificans* genome, and of doing so with a greater diversity of strains, 2 additional *L. prolificans* isolates were sequenced and these sequences, as well as those generated in Luo *et al*. (2017), were each assembled de novo. To confirm the reliability of the resulting assemblies, pulsed-field gel electrophoresis was conducted. Accordingly, we have produced 3 de novo whole genome assemblies, 1 to complete level and 2 to near-complete chromosome level. These chromosome lengths have been verified by pulsed-field gel electrophoresis.

## Materials and methods

### Strains

The strains used in this work were *L. prolificans* strains 3.1 from Christopher Thornton, ATCC strain 90853 and JHH-5317 from Sean Zhang. Conidia were obtained by inoculating frozen conidia into Sabouraud dextrose broth (BD, Franklin Lakes, NJ, USA), growing this at 30°C for 5–21 days shaking, then plating 2–3 mL onto potato dextrose agar. After 6–8 days of growth, these plates were covered in DPBS without magnesium or calcium and scraped. The resulting conidia in DPBS were strained through a 70 μm cell strainer, pelleted at 4000 RPM, washed 3 times, and stored at 4°C.

### DNA isolation

DNA was obtained from *L. prolificans* strains by growing cultures in Sabouraud dextrose broth shaking at 30°C. Fungus was collected by centrifugation and washed with sterile water, then incubated in DNA isolation buffer (0.1 M Tris-HCl, 0.2 NaCl, 5 mM EDTA, 0.2% SDS) with 1 mg/mL proteinase K at 55°C overnight. Following incubation, fungus was subjected to bead beating on a vortex with 0.5 mm zirconia/silica beads for 10 min. Samples were then centrifuged at 13,000 RPM for 10 min at room temperature. Supernatant was removed, added to an equal volume of 1:1 phenol chloroform, vortexed, and then centrifuged at 13,000 RPM for 5 min. The aqueous phase was transferred, added to an equal volume of chloroform, and vortexed. This was centrifuged at 13,000 RPM for 5 min, and the aqueous phase was removed. Twice the sample volume of molecular grade ethanol was added to the sample, which was then incubated at −20°C for at least 4 h. This was centrifuged at 13,000 RPM for 10 min at 4°C, following which the supernatant was discarded and 400 μL of 70% ethanol was added to the pellet. The sample was vortexed and then centrifuged at 13,000 RPM for 10 min at 4°C. Supernatant was discarded, and pellet was washed with 400 μL of 100% ethanol, and vortexing and centrifugation were repeated. Supernatant was discarded, and pellet was allowed to air dry before being resuspended in molecular grade water and stored at −20°C.

### DNA sequencing

Oxford Nanopore Technologies (ONT) sequencing libraries were prepared from genomic DNA using the Ligation Sequencing Kit (SQK-LSK109) with the Native Barcoding Kit (EXP-NBD103) according to the manufacturer’s specifications (Oxford Nanopore Technologies, Oxford, UK). Illumina sequencing libraries were prepared using the Nextera Flex DNA library prep kit (Illumina, San Diego, California) and sequenced on a MiSeq using v2 2×150 chemistry.

### Genome assembly and correction

ONT data were base-called and demultiplexed using Guppy v4.2.2 (Oxford Nanopore Technologies). Using reads greater than 3 kb long, 2 assemblies were generated, 1 using Canu v2.1.1 and 1 using Flye v2.9, both on default settings with the estimated genome size set to 39 Mb (Koren *et al*. 2017, 2018; Kolmogorov *et al*. 2019; Nurk *et al*. 2020). Nanopore reads were then aligned back to the assemblies using minimap2 v2.17. Alignments were used for correction with Racon v1.4.19 with settings -m 8 -x -6 -g -8 -w 500 (Vaser *et al*. 2017; Li 2018). Further corrections were then made using medaka v1.2.1 with the r941_min_high_g360 error model (medaka: Sequence correction provided by ONT Research.). Illumina reads were trimmed using Trimmomatic v0.39 with settings LEADING:3 TRAILING:3 SLIDINGWINDOW:4:30 MINLEN:36 (Bolger *et al*. 2014). Trimmed reads were then used to further correct the assemblies using Freebayes v1.3.4 in conjunction with the bowtie2 v2.4.2 aligner, both on default settings (Garrison and Marth 2012; Langmead and Salzberg 2012). Changes were made at loci where the alternative allele was both supported by more than 5 reads, and the alternative allele frequency was greater than 0.5. This Freebayes correction was performed iteratively until no changes could be made. To scaffold the contigs of the 2 corrected assemblies, Ragtag v1.0.2 was used on default settings (Alonge *et al*. 2022). For strain 3.1 and strain 90853, the corrected assembly generated by Flye was used as the reference for Ragtag, while for JHH-5317, the corrected assembly generated by Canu was used as the reference. The determination of the reference assembly was made based off of the telomere orientations of the resulting scaffolds.

Nanopore and trimmed Illumina reads were then aligned to the corrected assemblies using minimap2 and bowtie2, respectively, and contigs were broken at positions of 0 coverage unless within 1 kb of a contig end bearing telomere repeats (Langmead and Salzberg 2012; Li 2021). Mitochondrial contigs were identified with whole genome alignment to the *prolificans* reference genome from Luo *et al*. (2017) using Mummer v4.0.0 (Luo *et al*. 2017; Marçais *et al*. 2018). One contig containing repeats of the entire mitochondrial genome was trimmed to a single mitochondrial genome, while other contigs comprised of incomplete mitochondrial sequence were discarded. Lastly, the mean coverage for each contig was calculated with Nanopore and Illumina data separately. Contigs with coverage less than 90% of the median in both the Nanopore and Illumina data were discarded.

### Analysis

Genome analysis was performed in Geneious (Biomatters, Auckland, New Zealand). Genomes were aligned to one another using LASTZ with default parameters (Schwartz *et al*. 2003; Harris 2007). Subtelomeres were discovered by aligning the final 10 kb of each contig terminating with repeats of the telomeric sequence TTAGGG with all others from its strain-specific assembly using Geneious aligner. The resulting alignment was then edited by hand. Additional subtelomeres were then located by aligning the resulting consensus to the full genome assembly using LASTZ.

### Pulsed-field gel electrophoresis

Conidia were inoculated into Sabouraud dextrose broth, grown with shaking at 30°C for ∼16 h, and used to generate protoplasts using minor modifications of published methodology (Al-Laaeiby *et al*. 2016). Briefly, fungal biomass was filtered through Miracloth (Millipore Sigma, Burlington, MA), washed with sterile water, and incubated at 30°C on a nutating mixer for between 4 h and overnight in OM buffer with 5% lysing enzymes from *Trichoderma harzianum* (Millipore Sigma). Contents were then split into sterile centrifuge tubes and overlaid with chilled ST buffer in a ratio of 1.2 mL fungal solution to 1 mL ST buffer. Tubes were then centrifuged at 5,000 · g for 15 min at 4°C. Protoplasts were recovered at the interface of the 2 buffers and transferred to a sterile centrifuge tube, to which an equal volume of chilled STC buffer was added. Protoplasts were pelleted at 3000 g for 10 min at 4°C, following which supernatant was removed, and protoplasts were resuspended in 10 mL STC buffer. This was repeated 2 more times, with the final resuspension being performed with 200 mL GMB buffer. Plugs were then made and treated using methodology adapted from Brody and Carbon (Brody and Carbon 1989). Briefly, 200 mL of 1–2 × 10^9^ protoplasts in GMB buffer were mixed with 200 mL 2% low-melt agarose in 50 mM EDTA (pH 8) cooled to 42°C and pipetted into plug molds, which were then placed on ice for 10 min to solidify. Plugs were removed from the molds and incubated in NDS buffer with proteinase K at 50°C for 24 h, followed by 3 30 min washes in 50 mM EDTA (pH 8) at 50°C, then stored in 50 mM EDTA (pH 8) at 4°C.

Plugs were inserted into gels made with SeaKem Gold agarose (Lonza, Basel, Switzerland), along with *Saccharomyces cerevisiae*, *S. pombe*, or *Hansenula wingei* size standards (Bio-Rad Laboratories, Hercules, CA), and clamped homogeneous electrical field (CHEF) electrophoresis was run in TAE on a CHEF-DR III (Bio-Rad Laboratories). Gels were run at an included angle of 106° at 14°C according to the following conditions: 0.8% agarose at 3 V/cm with a switch time of 500 s for 48 h, at 14°C (Fig. 4a), 1% agarose at 2 V/cm with a switch time of 1,800 s for 100 h (Fig. 4b), and 0.8% agarose at 2 V/cm with a switch time of 1,800 s for 80 h (Fig. 4c).

After running, gels were stained with ethidium bromide and imaged using a ChemiDoc XRS+ gel imager (Bio-Rad Laboratories). Chromosome band lengths were estimated using Image Lab software (Bio-Rad Laboratories).

### RNA sequencing

Cultures were grown by inoculating conidia from strain JHH-5317 into 25 mL of RPMI to a concentration of 10^7^ conidia/mL, incubating the flasks at 37°C in 5% CO_2_ to facilitate germination for 4 h, then shaking them for 13 h at 37°C in atmospheric conditions. Following this, 25 mL of RPMI containing 16 μg/mL of ITR, 16 μg/mL of VRC, 8 μg/mL of AMB, or 2% DMSO by volume was added to each flask, for final concentrations of 8 μg/mL ITR, 8 μg/mL VRC, 4 μg/mL AMB, or 1% DMSO. Flasks were shaken at 37°C for 2 h, then strained through Whatman #2 filters (Cytiva, Marlborough, MA), washed twice with sterile Milli-Q water, added to 2 mL of TRIzol Reagent (Thermo Fisher Scientific, Waltham, MA), flash-frozen, and stored at −80°C. Three biological replicates were performed. All drugs were obtained from MilliporeSigma (Burlington, MA) and dissolved in DMSO.

Cell homogenization was performed in TRIzol Reagent using silica spheres (Lysing Matrix C, MP Biomedicals, Irvine, CA) in a FastPrep 120 (MP Biomedicals), with 4 intervals at speed 6 for 30 s. Homogenates remained on ice between shakes. Total RNA was purified using a PureLink RNA Mini Kit (Thermo Fisher Scientific), with the on-column PureLink DNase treatment, according to the manufacturer's instructions. RNA was quantified using a NanoDrop 1000. Quality assessment was performed by RNA ScreenTape Analysis in a TapeStation 2200 (Agilent Technologies, Santa Clara, CA).

Libraries for RNA-seq were prepared from 250 ng total RNA using the Illumina TruSeq Stranded mRNA Library Prep kit, according to the manufacturer's Low Sample protocol (Illumina, San Diego, CA). Quality assessment of libraries was conducted by High Sensitivity ScreenTape analysis on a TapeStation 2200 (Agilent Technologies). Libraries were quantified by qPCR with the Kapa Library Quantification kit (Roche, Basel, Switzerland) in a StepOne Plus Real-Time PCR System (Thermo Fisher Scientific). Libraries were diluted and pooled, and a final quality assessment was performed using High Sensitivity DNA LabChip Analysis on a BioAnalyzer 2100 (Agilent Technologies). A paired end, 2 × 100 bp, Illumina HiSeq 2500 run was performed at Johns Hopkins Genomics Genetic Resources Core Facility, RRID:SCR_018669.

### Genome annotation

RNA sequencing reads were aligned to each of the 3 genome assemblies using HISAT2 version 2.2.1 (Kim *et al*. 2019), and these alignments were input to Stringtie (Pertea *et al*. 2015) to produce transcript assemblies. We then aligned a collection of 8,390 Microascaceae family proteins available from GenBank, to each genome with NCBI tblastn version 2.13.0+, clustered the alignments and produced preliminary CDS features based on the local protein alignments with exonerate in protein2genome mode. We then filtered and reconciled the transcript and protein alignment features with gffcompare and gffread tools to produce final annotation in the GFF format. We then output the protein sequences and aligned them to the proteins in the UniProtKB database with blastp looking for a single best hit (accessed 01/2024). Any protein that had a significant (*e*-value < 10^−8^) hit was annotated as being similar to that protein in UniProtKB, and its function was assigned in the GFF file. BUSCO v5 was run on transcripts using gVolante (accessed 02/2024) (Humann *et al*. 2019; Manni *et al*. 2021).

## Results and discussion

Two strains of *L. prolificans*, strain 3.1 and strain 90853, were sequenced using both Nanopore and Illumina for long and short reads. These reads, as well as the reads from the sequencing of strain JHH-5317 used in Luo *et al*. (2017), were then separately assembled de novo, resulting in genomes of 36.77, 37.05, and 36.73 Mb long, with 18, 13, and 12 contigs, for JHH-5317, strain 3.1, and strain 90853, respectively (Table 1). Each assembly contains 1 circular contig of ∼24 kb that aligned to the mitochondrial genome of the previously published *L. prolificans* genome. Strains 3.1 and 90853, the former a soil isolate and the latter a clinical isolate that has been maintained in the laboratory for decades, were selected to complement JHH-5317, a clinical isolate, in the hopes of capturing a representative sample of the diversity of the species (Wood *et al*. 1992; Thornton *et al*. 2015).

**Table 1.** Length, contig number, and n50 of the 3 *L. prolificans* de novo whole genome assemblies.

Strain	Size	Contigs	n50
JHH-5317	36,771,655	18	4.584 Mb
Strain 3.1	37,053,789	13	3.387 Mb
Strain 90853	36,733,949	12	4.895 Mb

Of the 12 nonmitochondrial contigs in the strain 3.1 assembly, 10 have telomeric repeats (TTAGGG) on both ends, and the other 2 have telomeric repeats on one end (Table 2). The contigs range in size from 6.89 to 1.22 Mb. In the assembly of strain 90853, 9 of the 11 nonmitochondrial scaffolds have telomeric repeats on both ends, and another 2 have telomeric repeats on 1 end (Table 3). The contigs range in size from 5.69 to 1.22 Mb. Of the 17 nonmitochondrial contigs in the JHH-5317 assembly, none has telomeric repeats on both ends, and only 4 have telomeric repeats on one end (Table 4). These contigs range in size from 5.69 Mb to 7.27 kb, with 12 contigs longer than 1 Mb.

**Table 2.** Contigs of the genome assembly of strain 3.1.

Strain 3.1			
Contig name	Length (bases)	Forward telomeres*^a^*	Reverse telomeres*^a^*
**Contig beta**	**6,885,544**	**28**	**28**
Contig alpha-a	5,741,663	0	30
**Contig 5**	**3,850,082**	**32**	**24**
**Contig gamma**	**3,386,783**	**26**	**26**
**Contig delta**	**2,977,280**	**28**	**28**
**Contig 6**	**2,887,532**	**28**	**26**
**Contig 7**	**2,607,409**	**24**	**24**
**Contig 8**	**2,279,970**	**30**	**28**
Contig alpha-b	1,991,912	28	0
**Contig 9**	**1,876,168**	**24**	**32**
**Contig 10**	**1,322,933**	**26**	**24**
**Contig 11**	**1,222,527**	**26**	**26**
3.1 mito	23,986	0	0

Bolding indicates contigs with telomeres on both ends.

^a^Number of repeats of the telomeric sequence “TTAGGG” at the extreme end of each contig.

**Table 3.** Contigs of the genome assembly of strain 90853.

Strain 90853			
Contig name	Length (bases)	Forward telomeres*^a^*	Reverse telomeres*^a^*
Contig 1	5,688,415	30	0
Contig 2	5,146,484	0	40
**Contig 3**	**5,112,009**	**30**	**26**
**Contig 4**	**4,894,913**	**28**	**28**
**Contig 5**	**3,779,371**	**26**	**28**
**Contig 6**	**2,935,396**	**30**	**26**
**Contig 7**	**2,533,795**	**28**	**28**
**Contig 8**	**2,283,289**	**28**	**34**
**Contig 9**	**1,781,418**	**26**	**30**
**Contig 10**	**1,336,470**	**28**	**30**
**Contig 11**	**1,218,402**	**30**	**30**
90853 mito	23,987	0	0

Bolding indicates contigs with telomeres on both ends.

^a^Number of repeats of the telomeric sequence “TTAGGG” at the extreme end of each contig.

**Table 4.** Contigs of the genome assembly of strain JHH-5317.

JHH-5317					
Contig name	Length (bases)	Forward telomeres*^a^*	Reverse telomeres*^a^*	Forward subtelomere	Reverse subtelomere
*Contig 1*	*5,694,104*	*0*	*0*	*x*	*x*
*Contig 2*	*5,204,309*	*0*	*0*	*x*	*x*
*Contig 3*	*4,974,520*	*0*	*0*	*x*	*x*
Contig 4-a	4,583,919	0	0	x	
*Contig 5*	*3,804,892*	*0*	*18*	*x*	*x*
*Contig 7*	*2,601,742*	*0*	*2*	*x*	*x*
*Contig 8*	*2,282,424*	*10*	*0*	*x*	*x*
Contig 6-a	1,822,144	0	0	x	
*Contig 10*	1,273,626	*0*	*0*	*x*	*x*
*Contig 11*	1,235,459	*0*	*0*	*x*	*x*
Contig 9-a	1,217,717	0	0		x
Contig 6-b	1,114,990	0	0		x
Contig 9-b	365,328	0	0		
Contig 4-b	325,797	0	0	x	
Contig 9-c	224,199	0	2		x
5317 mito	24,023	0	0		
tig46	15,197	0	0		
Contig 36	7,265	0	0		

Italics indicates contigs with subtelomeres on both ends.

^a^Number of repeats of the telomeric sequence “TTAGGG” at the extreme end of each contig.

### Interstrain correspondence

**Fig. 1.**
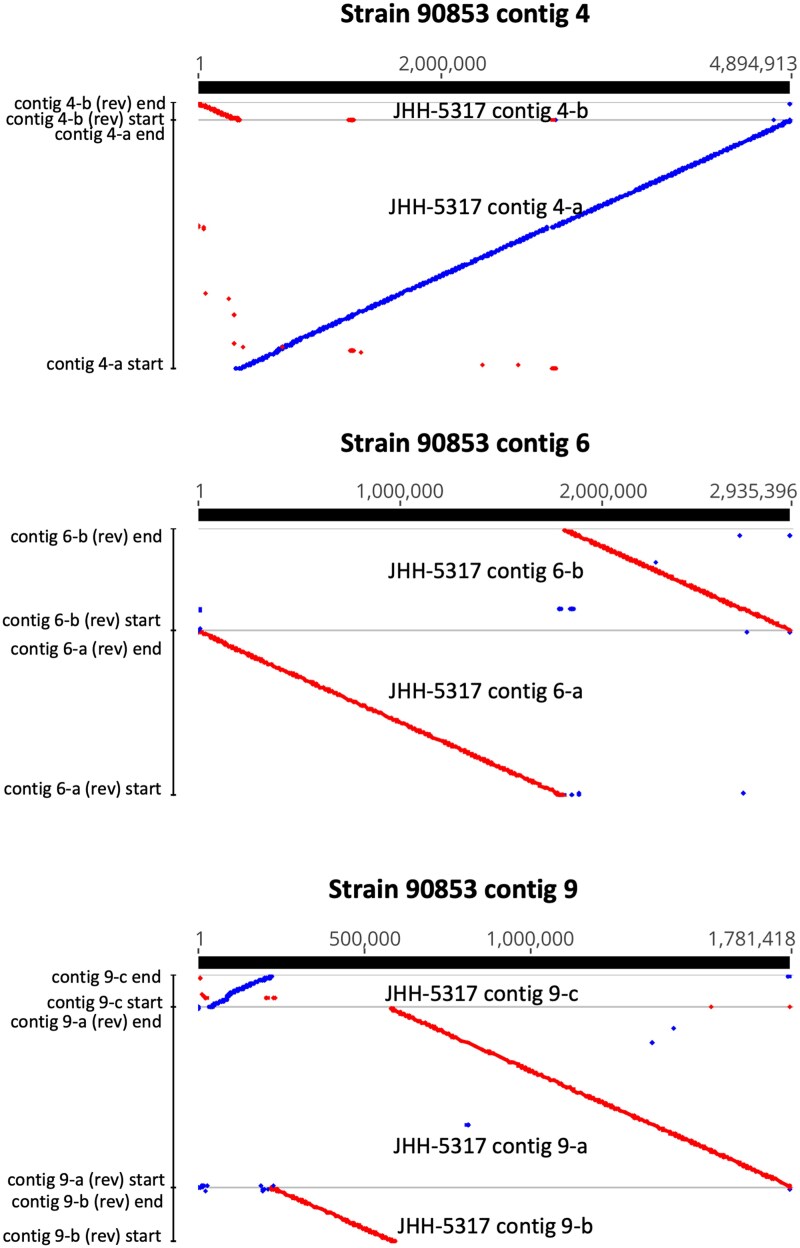
LASTZ alignments of JHH-5317 contigs representing fragmented chromosomes against 90853 contigs representing complete chromosomes. Blue indicates forward sequence orientation, and red indicates reverse sequence orientation.

To determine the correspondence of the genomes between the 3 strains, the assemblies were aligned to one another using LASTZ. As strain 90853 had the lowest number of contigs, these were used as the initial template and named contigs 1 through 11, starting with the longest contig. When the strain JHH-5317 assembly was mapped to strain 90853, there was 1 JHH-5317 contig mapped to 1 strain 90853 contig for strain 90853 contigs 1, 2, 3, 5, 7, 8, 10, and 11, and the matching JHH-5317 contigs were named accordingly. For strain 90853 contigs 4, 6, and 9, 2, 2, and 3 JHH-5317 contigs, respectively, mapped to each strain 90853 contig, and these were named contig 4-a and 4-b, contig 6-a and 6-b, and contig 9-a, 9-b, and 9-c (Fig. 1). Further evidence that contig 9-a and 9-b are in fact consecutive portions of the same chromosome is that, when mapped to strain 90853 contig 9, their meeting ends overlap approximately 11.6 kb with 96.8% pairwise identity. None of the other proposed consecutive contigs showed meaningful overlap.

The corresponding strain 90853 and JHH-5317 contigs showed high pairwise identity, ranging from 98.1 to 99.1%, and high coverage, ranging from 92.0 to 99.5% for coverage of strain 90853 contigs by JHH-5317 contigs and 96.1 to 99.4% for coverage of JHH-5317 contigs by strain 90853 contigs (Supplementary Table 1). The strains' mitochondrial genomes showed 99.4% pairwise identity and each mapped to the other for 99.1% coverage. The 2 smallest JHH-5317 contigs did not align clearly to any strain 90853 contigs; only 52 of the 7,265 bases of contig 36 mapped anywhere on the strain 90853 genome, while tig46 mapped to multiple places on each of the 11 contigs of strain 90853 and likely represents repeated sequence.

**Fig. 2.**
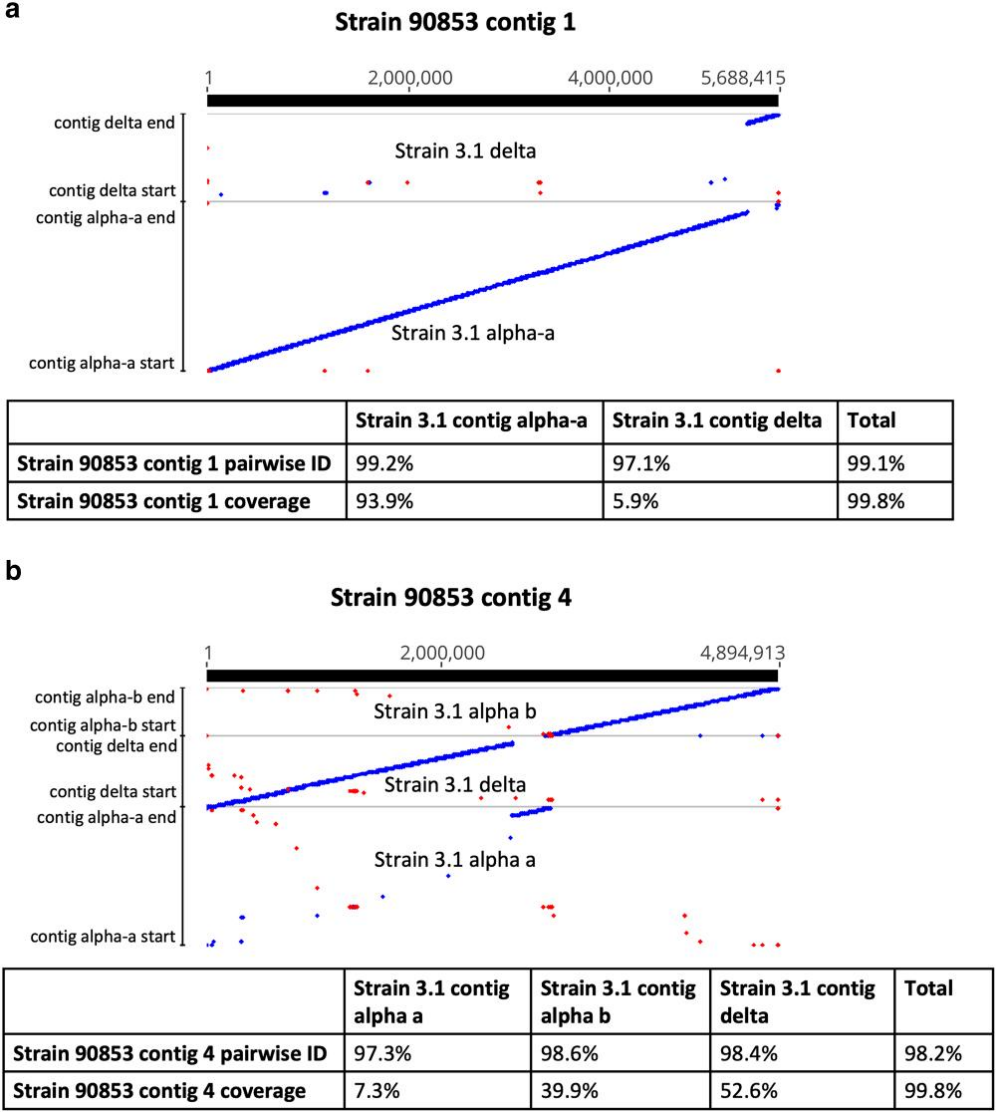
LASTZ alignments of strain 3.1 contigs alpha-a, alpha-b, and delta to strain 90853 a) contig 1 and b) contig 4. These contigs represent chromosomes that were involved in a chromosomal translocation. Pairwise identity and coverage are given below each alignment. Blue indicates forward sequence orientation, and red indicates reverse sequence orientation.

When strain 3.1 was mapped to strain 90853, 1 strain 3.1 contig each aligned to strain 90853 contigs 5 through 11, showing between 98.1 and 99.1% pairwise identity, between 91.4 and 99.6% coverage of strain 90853 contigs by strain 3.1 contigs, and between 91.1 and 98.6% coverage of strain 3.1 contigs by strain 90853 contigs (Supplementary Table 1). The mitochondrial genomes showed 99.97% pairwise identity and 99.9% coverage for both strains. However, when the strain 3.1 assembly was mapped to strain 90853 contigs 1 through 4, evidence of a chromosomal translocation was observed. The first 5.4 Mb of strain 3.1 contig alpha-a mapped to the first 5.4 Mb of strain 90853 contig 1, but the remaining 0.32 Mb of strain 90853 contig 1 aligned to the last 0.32 Mb of strain 3.1 contig delta (Fig. 2). The first 2.6 Mb of strain 3.1 contig delta mapped to the first 2.6 Mb of strain 90853 contig 4, followed by the final 0.33 Mb of strain 3.1 contig alpha-a, and then the full 1.9 Mb of strain 3.1 contig alpha-b. This suggests an event in which strain 90853 contigs 1 and 4 each broke into 2 pieces and the 4 resulting pieces were incorrectly repaired, resulting in strain 3.1 contigs alpha-a and alpha-b, likely pieces of the same chromosome, and strain 3.1 contig delta.

These breakpoints are further supported by long read alignments, where strain 90853 Nanopore reads aligned to contig 1 of the strain 90953 assembly showed no aberrant coverage near the breakpoint, while strain 3.1 Nanopore reads aligned to the strain 90853 assembly showed coverage drops indicative of a rearrangement. Similarly, when Nanopore reads of strain 3.1 are aligned to contig delta of the strain 3.1 assembly, no aberrant coverage patterns are observed, while alignment of strain 90853 Nanopore reads to contig delta of the strain 3.1 assembly resulted in a coverage gap suggesting a structural variation exists at that locus. Taken together, the read alignment evidence argues against the possibility that these contig rearrangements are the result of any misassembly. The hypothesis that contigs alpha-a and alpha-b are 2 pieces of the same chromosome that were not joined by the assembler in silico is supported by the fact that the meeting ends of these 2 contigs share 15.1 kb of overlap with 96.8% pairwise identity. Additionally, each of the contigs has telomeric repeats only on its nonoverlapping end, while all the other nonmitochondrial contigs of the strain 3.1 assembly have telomeric repeats on both ends and are, accordingly, likely to be complete chromosomes.

**Fig. 3.**
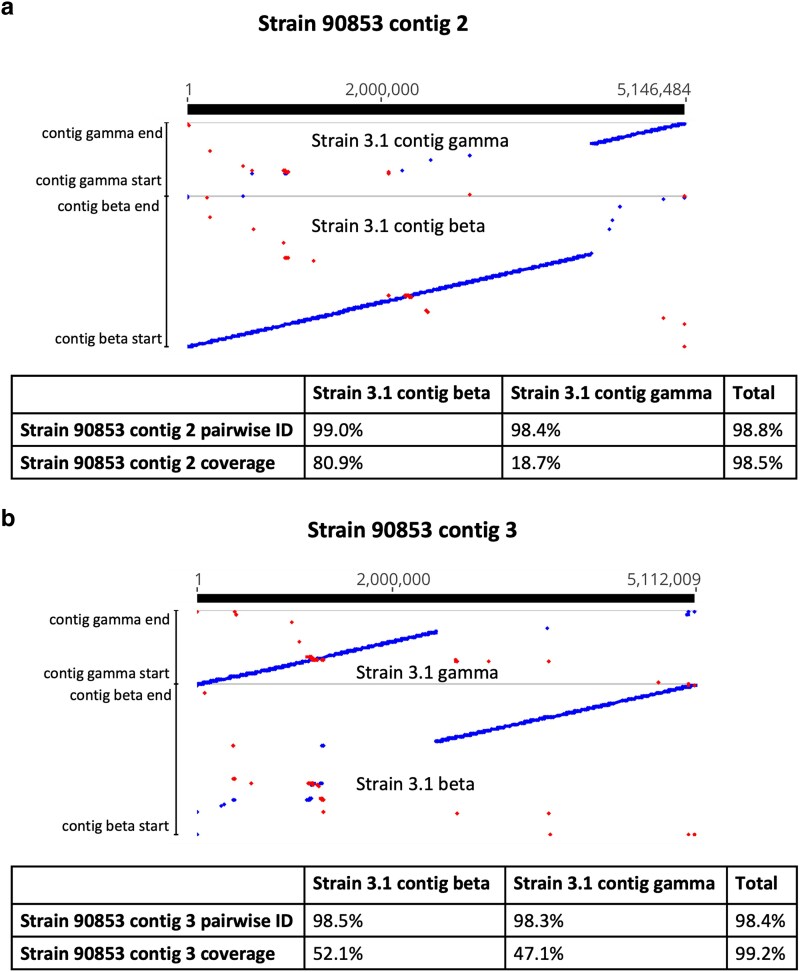
LASTZ alignments of strain 90853 a) contig 2 and b) contig 3 and strain 3.1 contigs beta and gamma to one another. These contigs represent chromosomes that were involved in a chromosomal translocation. Pairwise identity and coverage are given below each alignment. Blue indicates forward sequence orientation, and red indicates reverse sequence orientation.

A similar reconfiguration was seen with strain 90853 contigs 2 and 3 and strain 3.1 contigs beta and gamma, in which strain 3.1 contig beta seems to be made up of the first 4.2 Mb of strain 90853 contig 2 and the last 2.7 Mb of strain 90853 contig 3. Strain 3.1 contig gamma, likewise, is made up of the first 2.5 Mb of strain 90853 contig 3 and the last 1.0 Mb of strain 90853 contig 2 (Fig. 3). These breakpoints are also supported by Nanopore alignment evidence.

### Subtelomeres

To determine whether the subtelomeric region of the *L. prolificans* genome has shared characteristics across chromosome and strains, the final 10 kb of contig ends containing telomeric repeats were aligned by strain. In strains JHH-5317 and 3.1, all putative chromosome ends aligned with one another, resulting in consensus sequences 8,271 and 7,590 bases long with 95.4 and 95.8% pairwise identity, respectively (Supplementary Files 1 and 2).

No single sequence was conserved across the 20 putative chromosome ends of strain 90853, but 3 sequences were conserved across several putative chromosome ends each (Supplementary Files 3–5 and Table 2). The shortest of these, consensus region 3, is, with some variation, the reverse complement sequence of a section of the longest, consensus region 1.

When each of the 3 strains' conserved subtelomeric sequences was aligned to one another, those of strain 3.1 and JHH-5317 showed 97.8% pairwise identity, while strain 90853 subtelomeric consensus region 2, shared by 7 putative chromosome ends, mapped to both the strain 3.1 and JHH-5317 sequences. The remaining subtelomeric sequences of strain 90853 did not map to those of the other 2 strains.

The presence of highly conserved subtelomeric sequences presented the opportunity to test subtelomeres as indicators of complete chromosomes on contigs where telomeres are not present. Accordingly, consensus sequences generated from each strain's subtelomere alignment were mapped against that strain's whole genome. In strain 3.1, this sequence mapped to each contig end that contained telomeres and nowhere else in the genome. In strain 90853, subtelomeric consensus sequence 2 mapped exclusively to contig ends that contained telomeres, but only to 8 of the total 20, which was in keeping with the lower level of subtelomeric conservation seen in this strain's genome compared to the other 2 strains. The specificity of this conserved subtelomeric sequence to telomere-adjacent areas of the genome demonstrates that, in the absence of telomeres, searching for subtelomeric sequences in *L. prolificans* genomic assemblies is likely to be a specific, though not always a sensitive, means of identifying chromosome ends.

Mapping strain 90853 subtelomeric consensus region 1 back onto the assembly of strain 90853 produced less consistent results. Overall, it mapped to 14 regions in 10 contigs that were within 20 kb of contig end and 12 regions more than 20 kb from an end. Accordingly, consensus with this sequence appears to be predictive of a subtelomeric region, but it is not unique to subtelomeres.

The principle of identifying chromosome ends using subtelomeric sequences was utilized for the JHH-5317 assembly, which contains only 4 clusters of telomeric repeats. By mapping the conserved JHH-5317 subtelomere sequence to the full assembly, 22 regions containing portions of the sequence were revealed, all located at the beginning or end of a contig. These were distributed such that the 8 contigs that corresponded one-to-one with a full strain 90853 contig contigs contained subtelomeres on both ends, the 6 contigs that mapped to just one end of a full strain 90853 contig contained a subtelomere on just one end, and neither the 2 smallest contigs nor contig 9-b, which mapped to the middle of strain 90853 contig 9, had any subtelomeres. This provides compelling evidence for the correctness of genome assembly, given that subtelomeric sequences mapped only to the extreme ends of contigs. Additionally, it supports the identification of contigs or contig groups as chromosomes established through mapping the JHH-5317 contigs to the strain 90853 assembly.

### Pulsed-field gel electrophoresis

**Fig. 4.**
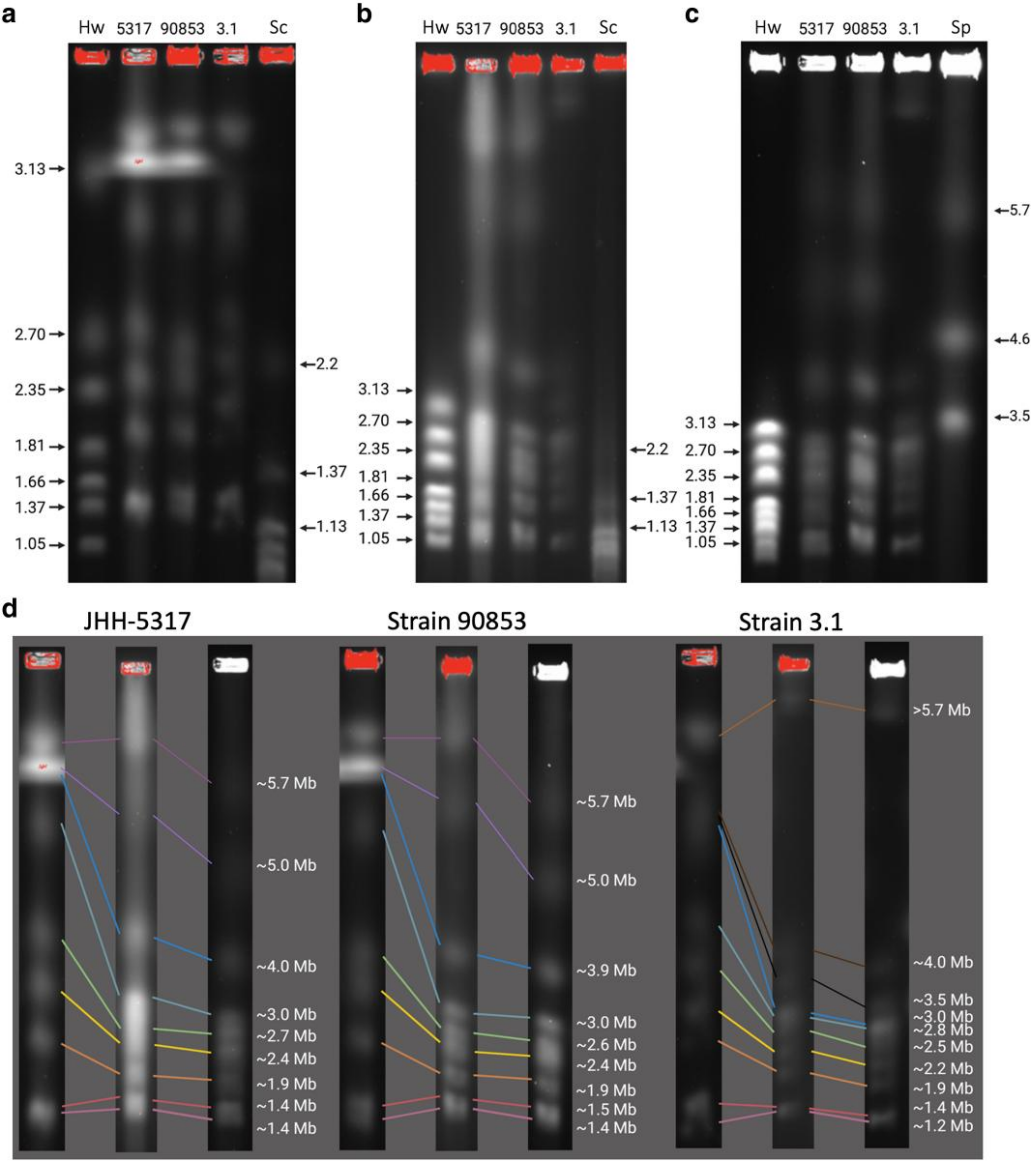
Pulsed-field gel electrophoresis of *L. prolificans* strains. a–c) Chromosomal DNA from *H. wingei* (Hw), S. cerevisiae (Sc), and *S. pombe* (Sp), and *L. prolificans* strains JHH-5317, ATCC 90853, and 3.1 were run under 3 different conditions. d) Comparison of *L. prolificans* chromosomal bands with standard bands of known length allowed the estimation of the lengths of the *L. prolificans* chromosomes. See Materials and methods for condition details. Made using Biorender.com.

To provide physical corroboration for the insights provided by the computational analysis of sequence data, pulsed-field gel electrophoresis was performed for the 3 sequenced strains, thereby visualizing their chromosomes. Examination of the resulting images shows 9 bands for strains 90853 and JHH-5317 ranging from 1.4 to 5.7 Mb in length and 10 bands for strain 3.1 ranging from 1.2 to much greater than 5.7 Mb (above which estimation is not possible, as the longest ladder extends only up to 5.7 MB) in length (Fig. 4). The sum of the lengths of bands comes to 27.4, 27.5, and 28.2 Mb for strains 90853, JHH-5317, and 3.1 (counting the large band of unknown size as 5.7 Mb), respectively. Given that these lengths are all approximately 9 Mb shorter than the lengths of the sequenced genomes, it is likely that at least 1 of these bands represents multiple chromosomes.

It is notable that while strains 90853 and JHH-5317 showed roughly the same banding pattern, strain 3.1 differed notably, with a band around 3.5 Mb where strain 90853 and JHH-5317 show none, and no bands longer than 4.6 Mb until the longest band, high above 5.7 Mb, while strain 90853 and JHH-5317 have 3 bands in this range (Table 5). This corresponds to the chromosome sizes observed in the genome assemblies and confirms that the chromosomal translocation of strain 90853 contigs 1 through 4 into strain 3.1 contigs alpha through delta has actually occurred, resulting in chromosomes of distinctly different lengths.

**Table 5.** Correspondence of chromosome lengths in *L. prolificans* genome assemblies with chromosome lengths estimated by pulsed-field gel electrophoresis.

	Strain 90853		JHH-5317		Strain 3.1		
Chromosome	Length (Mb)	Matching band	Length (Mb)	Matching band	Length (Mb)	Matching band	Chromosome
**1**	5.69	∼5.7	5.69	∼5.7	7.73	>>5.7	**Alpha**
**2**	5.15	∼5	5.20	∼5	6.89	>>5.7	**Beta**
**3**	5.11	∼5	4.97	∼5	3.85	∼4	**5**
**4**	4.89	∼5	4.91	∼5	3.39	∼3.5	**Gamma**
**5**	3.78	∼3.9	3.80	∼4	2.98	∼3	**Delta**
**6**	2.94	∼3	2.94	∼3	2.89	∼2.8	**6**
**7**	2.53	∼2.6	2.60	∼2.7	2.61	∼2.5	**7**
**8**	2.28	∼2.4	2.28	∼2.4	2.28	∼2.2	**8**
**9**	1.78	∼1.9	1.81	∼1.9	1.88	∼1.9	**9**
**10**	1.34	∼1.5	1.27	∼1.4	1.32	∼1.4	**10**
**11**	1.22	∼1.4	1.24	∼1.4	1.22	∼1.2	**11**

Shading indicates the corresponding chromosome names for each strain, with strains 90853 and JHH-5317 corresponding to the leftmost column, and strain 3.1 corresponding to the rightmost column.

### Genome completeness

To estimate the completeness of the 3 genome assemblies, BUSCO was used to search them for orthologs to the 3817 gene groups expected to be present as single-copy genes in *Sordariomycetes* (based on those found as such in ≥90% of the phylum) (Manni *et al*. 2021). The results were similar across the 3 assemblies, with 95.3–95.7% (3,636–3,653) of expected genes present as single copies, 0.5% (18–19) present in duplicate, 0.7–1.0% (26–37) present in a fragmented form, and 3.1–3.3% (120–125) missing entirely (Table 6). This represents an improvement from the first published *L. prolificans* genome assembly that when compared to a set of 3,725 expected gene groups, found 94.2% present either singly or in duplicate (Luo *et al*. 2017). Of the missing gene groups, 109 were shared across all 3 genomes. It is therefore likely that many of the “missing” gene groups are reflective of true absence from the *L. prolificans* genome, rather than failures in the sequencing or assembly.

**Table 6.** Results of BUSCO search of *L. prolificans* genome assembles for single-copy genes expected in *Sordariomycetes*.

	JHH-5317	Strain 90853	Strain 3.1
**Complete**	3655 (95.6%)	3,671 (96.2%)	3,669 (96.1%)
** Single**	3,636 (95.3%)	3,653 (95.7%)	3,650 (95.6%)
** Duplicated**	19 (0.5%)	18 (0.5%)	19 (0.5%)
**Fragmented**	37 (1.0%)	26 (0.7%)	27 (0.7%)
**Missing**	125 (3.3%)	120 (3.1%)	121 (3.2%)

### Annotation

The assemblies, in combination with ∼16.1 Gbp of sequence in Illumina RNA-seq reads from JHH-5317 and a set of 8,390 proteins from the family Microascaceae, were used as evidence to generate gene annotation for the 3 *L. prolificans* strains (Supplementary Files 6–8). The results are reported in Supplementary Table 3. These transcripts and the set of proteins were then used to produce annotations for the 3 strains, resulting in between 7,559 and 7,898 transcripts per strain and between 6,927 and 7,180 genes per strain (Supplementary Table 4). The BUSCO results of the transcripts are similar across the 3 strains, with 88.4–89.7% of expected genes appearing complete, ∼0.8–1.3% appearing in fragmented forms, and 9.2–10.3% missing (Supplementary Table 5).

## Conclusions

In this work, we determined that *L. prolificans* possesses 11 nuclear chromosomes made up of 36.7–37.1 Mb with 7,357–7,640 genes identified in each strain. The evidence of the telomeres, subtelomeres, and pulsed-field gel electrophoresis provides compelling evidence that we have produced a complete-level assembly of *L. prolificans* strain ATCC 90853 and very nearly complete chromosome-level assemblies of strains JHH-5317 and strain 3.1. The BUSCO results show >95% of expected single-copy genes present in their complete forms, and of the 120–125 genes missing in the 3 strains, 109 are missing across all 3, suggesting that a large portion of the missing genes are genuinely absent from the *L. prolificans* genome. Together, this supports the conclusion that these genome assemblies are complete.

The discovery of an apparent chromosomal translocation event in 1 of the 3 strains is intriguing. Assessments of the diversity of *L. prolificans* have varied (Rainer *et al*. 2000; Solé *et al*. 2003; Delhaes *et al*. 2008; Harun *et al*. 2009; Lackner and de Hoog 2011; Lackner *et al*. 2014) and would benefit from the sequencing of more strains to elucidate the species structure and provide a greater understanding of how anomalous this chromosomal translocation is in this species.

We believe the availability of complete, diverse, and annotated chromosome-level genome assemblies of *L. prolificans* will be a valuable resource from which many new insights into *L. prolificans* biology can be gained.

## Supplementary Material

jkaf091_Supplementary_Data

## Data Availability

All sequence data are available in the Sequence Read Archive and GenBank, under BioProject PRJNA929059. All code used for analysis can be found at https://github.com/timplab/grossman_prolificans. Annotation Supplementary Files 6–8 are available on GSA FigShare at https://doi.org/10.25387/g3.28836872. Supplemental material available at G3 online.

## References

[jkaf091-B1] Al-Laaeiby A, Kershaw MJ, Penn TJ, Thornton CR. 2016. Targeted disruption of melanin biosynthesis genes in the human pathogenic fungus Lomentospora prolificans and its consequences for pathogen survival. Int J Mol Sci. 17(4):444. doi:10.3390/ijms17040444. 27023523 PMC4848900

[jkaf091-B2] Alonge M, Lebeigle L, Kirsche M, Jenike K, Ou S, Aganezov S, Wang X, Lippman ZB, Schatz MC, Soyk S. 2022. Automated assembly scaffolding using RagTag elevates a new tomato system for high-throughput genome editing. Genome Biol. 23(1):258. doi:10.1186/s13059-021-02568-9. 36522651 PMC9753292

[jkaf091-B3] Bolger AM, Lohse M, Usadel B. 2014. Trimmomatic: a flexible trimmer for Illumina sequence data. Bioinformatics. 30(15):2114–2120. doi:10.1093/bioinformatics/btu170. 24695404 PMC4103590

[jkaf091-B4] Brody H, Carbon J. 1989. Electrophoretic karyotype of Aspergillus nidulans. Proc Natl Acad Sci U S A. 86(16):6260–6263. doi:10.1073/pnas.86.16.6260. 2668960 PMC297817

[jkaf091-B5] Delhaes L, Harun A, Chen SC, Nguyen Q, Slavin M, Heath CH, Maszewska K, Halliday C, Robert V, Sorrell TC, et al 2008. Molecular typing of Australian Scedosporium isolates showing genetic variability and numerous S. aurantiacum. Emerg Infect Dis. 14(2):282–290. doi:10.3201/eid1402.070920. 18258122 PMC2600218

[jkaf091-B6] Garrison E, Marth G. 2012. Haplotype-based variant detection from short-read sequencing. arXiv 1207.3907. 10.48550/arXiv.1207.3907, preprint: not peer reviewed.

[jkaf091-B7] Harris RS . 2007. Improved pairwise alignment of genomic DNA. [accessed 2021 Jul]. https://www.bx.psu.edu/~rsharris/rsharris_phd_thesis_2007.pdf.

[jkaf091-B8] Harun A, Perdomo H, Gilgado F, Chen SC, Cano J, Guarro J, Meyer W. 2009. Genotyping of Scedosporium species: a review of molecular approaches. Med Mycol. 47(4):406–414. doi:10.1080/13693780802510240. 19085455

[jkaf091-B9] Humann JL, Lee T, Ficklin S, Main D. 2019. Structural and functional annotation of eukaryotic genomes with GenSAS. Methods Mol Biol. 1962:29–51. doi:10.1007/978-1-4939-9173-0_3 31020553

[jkaf091-B10] Jacobs SE, Wengenack NL, Walsh TJ. 2020. Non-Aspergillus hyaline molds: emerging causes of sino-pulmonary fungal infections and other invasive mycoses. Semin Respir Crit Care Med. 41(1):115–130. doi:10.1055/s-0039-3401989. 32000288

[jkaf091-B11] Jenks JD, Seidel D, Cornely OA, Chen S, van Hal S, Kauffman C, Miceli MH, Heinemann M, Christner M, Jover Sáenz A, et al 2020. Clinical characteristics and outcomes of invasive Lomentospora prolificans infections: analysis of patients in the FungiScope® registry. Mycoses. 63(5):437–442. doi:10.1111/myc.13067. 32080902

[jkaf091-B12] Kim D, Paggi JM, Park C, Bennett C, Salzberg SL. 2019. Graph-based genome alignment and genotyping with HISAT2 and HISAT-genotype. Nat Biotechnol. 37(8):907–915. doi:10.1038/s41587-019-0201-4. 31375807 PMC7605509

[jkaf091-B13] Kolmogorov M, Yuan J, Lin Y, Pevzner PA. 2019. Assembly of long, error-prone reads using repeat graphs. Nat Biotechnol. 37(5):540–546. doi:10.1038/s41587-019-0072-8. 30936562

[jkaf091-B14] Koren S, Rhie A, Walenz BP, Dilthey AT, Bickhart DM, Kingan SB, Hiendleder S, Williams JL, Smith TPL, Phillippy AM. 2018. De novo assembly of haplotype-resolved genomes with trio binning. Nat Biotechnol. 36(12):1174–1182. doi:10.1038/nbt.4277. PMC6476705 30346939

[jkaf091-B15] Koren S, Walenz BP, Berlin K, Miller JR, Bergman NH, Phillippy AM. 2017. Canu: scalable and accurate long-read assembly via adaptive *κ*-mer weighting and repeat separation. Genome Res. 27(5):722–736. doi:10.1101/gr.215087.116. 28298431 PMC5411767

[jkaf091-B16] Lackner M, de Hoog GS. 2011. Parascedosporium and its relatives: phylogeny and ecological trends. IMA Fungus. 2(1):39–48. doi:10.5598/imafungus.2011.02.01.07. 22679587 PMC3317366

[jkaf091-B17] Lackner M, de Hoog GS, Yang L, Ferreira Moreno L, Ahmed SA, Andreas F, Kaltseis J, Nagl M, Lass-Flörl C, Risslegger B, et al 2014. Proposed nomenclature for Pseudallescheria, Scedosporium and related genera. Fungal Divers. 67(1):1–10. doi:10.1007/s13225-014-0295-4.

[jkaf091-B18] Langmead B, Salzberg SL. 2012. Fast gapped-read alignment with Bowtie 2. Nat Methods. 9(4):357–359. doi:10.1038/nmeth.1923. 22388286 PMC3322381

[jkaf091-B19] Li H . 2018. Minimap2: pairwise alignment for nucleotide sequences. Bioinformatics. 34(18):3094–3100. doi:10.1093/bioinformatics/bty191. 29750242 PMC6137996

[jkaf091-B20] Li H . 2021. New strategies to improve minimap2 alignment accuracy. Bioinformatics. 37(23):4572–4574. doi:10.1093/bioinformatics/btab705. 34623391 PMC8652018

[jkaf091-B21] Luo R, Zimin A, Workman R, Fan Y, Pertea G, Grossman N, Wear MP, Jia B, Miller H, Casadevall A, et al 2017. First draft genome sequence of the pathogenic fungus *Lomentospora prolificans* (formerly *Scedosporium prolificans*). G3 (Bethesda). 7(11):3831–3836. doi:10.1534/g3.117.300107. 28963165 PMC5677167

[jkaf091-B22] Manni M, Berkeley MR, Seppey M, Simão FA, Zdobnov EM. 2021. BUSCO update: novel and streamlined workflows along with broader and deeper phylogenetic coverage for scoring of eukaryotic, prokaryotic, and viral genomes. Mol Biol Evol. 38(10):4647–4654. doi:10.1093/molbev/msab199. 34320186 PMC8476166

[jkaf091-B23] Marçais G, Delcher AL, Phillippy AM, Coston R, Salzberg SL, Zimin A. 2018. MUMmer4: a fast and versatile genome alignment system. PLoS Comput Biol. 14(1):e1005944. doi:10.1371/journal.pcbi.1005944. 29373581 PMC5802927

[jkaf091-B24] medaka: Sequence correction provided by ONT Research . [accessed 2021 Jul]. https://github.com/nanoporetech/medaka.

[jkaf091-B25] Nurk S, Walenz BP, Rhie A, Vollger MR, Logsdon GA, Grothe R, Miga KH, Eichler EE, Phillippy AM, Koren S. 2020. Hicanu: accurate assembly of segmental duplications, satellites, and allelic variants from high-fidelity long reads. Genome Res. 30(9):1291–1305. doi:10.1101/gr.263566.120. 32801147 PMC7545148

[jkaf091-B26] Pertea M, Pertea GM, Antonescu CM, Chang TC, Mendell JT, Salzberg SL. 2015. StringTie enables improved reconstruction of a transcriptome from RNA-Seq reads. Nat Biotechnol. 33(3):290–295. doi:10.1038/nbt.3122. 25690850 PMC4643835

[jkaf091-B27] Rainer J, De Hoog GS, Wedde M, Gräser Y, Gilges S. 2000. Molecular variability of Pseudallescheria boydii, a neurotropic opportunist. J Clin Microbiol. 38(9):3267–3273. doi:10.1128/JCM.38.9.3267-3273.2000. 10970369 PMC87372

[jkaf091-B28] Schwartz S, Kent WJ, Smit A, Zhang Z, Baertsch R, Hardison RC, Haussler D, Miller W. 2003. Human-mouse alignments with BLASTZ. Genome Res. 13(1):103–107. doi:10.1101/gr.809403. 12529312 PMC430961

[jkaf091-B29] Solé M, Cano J, Rodríguez-Tudela JL, Pontón J, Sutton DA, Perrie R, Gené J, Rodríguez V, Guarro J. 2003. Molecular typing of clinical and environmental isolates of Scedosporium prolificans by inter-simple-sequence-repeat polymerase chain reaction. Med Mycol. 41(4):293–300. doi:10.1080/13693780310001600813. 12964722

[jkaf091-B30] Thornton CR, Ryder LS, Le Cocq K, Soanes DM. 2015. Identifying the emerging human pathogen Scedosporium prolificans by using a species-specific monoclonal antibody that binds to the melanin biosynthetic enzyme tetrahydroxynaphthalene reductase. Environ Microbiol. 17(4):1023–1038. doi:10.1111/1462-2920.12470. 24684242

[jkaf091-B31] Vaser R, Sović I, Nagarajan N, Šikić M. 2017. Fast and accurate de novo genome assembly from long uncorrected reads. Genome Res. 27(5):737–746. doi:10.1101/gr.214270.116. 28100585 PMC5411768

[jkaf091-B32] Wood GM, McCormack JG, Muir DB, Ellis DH, Ridley MF, Pritchard R, Harrison M. 1992. Clinical features of human infection with Scedosporium inflatum. Clin Infect Dis. 14(5):1027–1033. doi:10.1093/clinids/14.5.1027. 1534693

[jkaf091-B33] Wu Y, Grossman N, Totten M, Memon W, Fitzgerald A, Ying C, Zhang SX. 2020. Antifungal susceptibility profiles and drug resistance mechanisms of clinical lomentospora prolificans isolates. Antimicrob Agents Chemother. 64(11):e00318-20. doi:10.1128/AAC.00318-20. 32816726 PMC7577128

