# Supplementary material for: Three annotated chromosome-level de novo genome assemblies of Lomentospora prolificans provide evidence for a chromosomal translocation event
Source: G3 (Bethesda). 2025 Apr 24;15(6):jkaf091. doi: 10.1093/g3journal/jkaf091 (PMC12134988; doi:10.1093/g3journal/jkaf091)
Supplement: jkaf091_Supplementary_Data [file jkaf091_supplementary_data.zip › Tables_S1-S5_G3-2025-405647.docx]

**Table S1:** Similarity of three *L. prolificans* genome assemblies across orthologous contigs. NA where chromosomal translocation prevents one-to-one comparison. PI: pairwise identity, cov.: coverage, NA: not applicable.

|  | **Strain 90853 vs Strain 3.1** | | | | **Strain 90853 vs JHH-5317** | | | | **Strain 3.1 vs** **JHH-5317** | | | |
| --- | --- | --- | --- | --- | --- | --- | --- | --- | --- | --- | --- | --- |
|  | **Strain 90853** | | **Strain 3.1** | | **Strain 90853** | | **JHH-5317** | | **Strain 3.1** | | **JHH-5317** | |
|  | **%PI** | **%cov.** | **%PI** | **%cov.** | **%PI** | **%cov.** | **%PI** | **%cov.** | **%PI** | **%cov.** | **%PI** | **%cov.** |
| **Contig 1** | NA | NA | NA | NA | 99.0 | 98.9 | 99.0 | 98.9 | NA | NA | NA | NA |
| **Contig 2** | NA | NA | NA | NA | 99.0 | 99.5 | 99.0 | 99.4 | NA | NA | NA | NA |
| **Contig 3** | NA | NA | NA | NA | 98.8 | 96.9 | 98.7 | 98.9 | NA | NA | NA | NA |
| **Contig 4** | NA | NA | NA | NA | 98.6 | 97.7 | 98.7 | 97.5 | NA | NA | NA | NA |
| **Contig 5** | 99.1 | 98.7 | 99.1 | 97.2 | 99.1 | 98.5 | 99.1 | 97.9 | 99.1 | 98.6 | 99.1 | 99.4 |
| **Contig 6** | 98.8 | 96.6 | 98.8 | 98.0 | 98.6 | 98.7 | 98.7 | 98.4 | 98.7 | 98.6 | 98.7 | 97.1 |
| **Contig 7** | 98.8 | 99.6 | 98.9 | 97.2 | 98.8 | 98.9 | 98.8 | 96.5 | 98.9 | 99.2 | 98.9 | 99.2 |
| **Contig 8** | 98.6 | 97.6 | 98.8 | 98.0 | 98.8 | 98.0 | 98.8 | 98.1 | 99.3 | 99.2 | 99.3 | 99.1 |
| **Contig 9** | 98.6 | 96.3 | 98.5 | 91.1 | 98.4 | 97.4 | 98.4 | 97.0 | 98.8 | 93.2 | 98.9 | 97.7 |
| **Contig 10** | 98.1 | 91.4 | 98.0 | 92.9 | 98.1 | 92.0 | 98.0 | 96.1 | 98.9 | 95.3 | 98.9 | 98.6 |
| **Contig 11** | 98.9 | 99.0 | 98.9 | 98.6 | 98.8 | 99.2 | 98.8 | 98.1 | 98.8 | 98.5 | 98.8 | 98.5 |
| **mito** | 99.97 | 99.9 | 99.97 | 99.9 | 99.4 | 99.1 | 99.4 | 99.1 | 99.4 | 99.3 | 99.4 | 99.2 |

**Table S2:** Subtelomeric consensus regions from the genome assembly of strain 90853.

| **Strain 90853 consensus region** | **# chromosome ends** | **Length (bases)** | **pairwise identity** |
| --- | --- | --- | --- |
| **1** | 11 | 7,251 | 72.7% |
| **2** | 7 | 3,901 | 82.3% |
| **3** | 8 | 3,160 | 86.3% |

**Table S3.** Assembled transcript statistics for *L. prolifcans* genomes.

| **Strain** | **JHH-5317** | **Strain 90853** | **Strain 3.1** |
| --- | --- | --- | --- |
| **Number of transcripts** | 11640 | 12219 | 11930 |
| **N50 size of transcripts** | 4630 | 4185 | 4345 |
| **Total sequence in transcripts** | 37,058,901 | 36,362,687 | 36,441,817 |

**Table S4.** Annotation statistics for *L. prolifcans* genomes.

| **Strain** | **JHH-5317** | **Strain 90853** | **Strain 3.1** |
| --- | --- | --- | --- |
| **Number of transcripts** | 7559 | 7898 | 7755 |
| **Number of genes** | 7357 | 7640 | 7530 |
| **Number of single-exon genes** | 440 | 552 | 518 |
| **Number of putative protein coding genes** | 4949 | 4990 | 4964 |
| **Number of protein coding functionally annotated with UniProt alignments** | 4153 | 4195 | 4172 |
| **Number of all protein coding genes** | 6927 | 7180 | 7104 |
| **Alternative splicing** | | | |
| **Number of genes with 1 isoform** | 5820 | 6081 | 5981 |
| **Number of genes with 2 isoforms** | 917 | 946 | 957 |
| **Number of genes with 3 isoforms** | 387 | 384 | 360 |
| **Number of genes with 4 isoforms** | 131 | 125 | 136 |
| **Number of genes with >4 isoforms** | 102 | 104 | 96 |

**Table S5.** BUSCO results for *L. prolificans* transcripts

| **Strain** | **JHH-5317** | **Strain 90853** | **Strain 3.1** |
| --- | --- | --- | --- |
| **Complete**  **Single**  **Duplicated** | 3362 (88.4%) | 3425 (89.7%) | 3423 (89.7%) |
|  | 2994 (78.4%) | 3057 (80.1%) | 3040 (79.6%) |
|  | 379 (9.9%) | 368 (9.6%) | 383 (10.0%) |
| **Fragmented** | 50 (1.3%) | 39 (1.0%) | 30 (0.8%) |
| **Missing** | 394 (10.3%) | 353 (9.2%) | 364 (9.5%) |
